# Perspective on pH adjustment in hydrometallurgical recycling of valuable metals from waste

**DOI:** 10.3389/fchem.2023.1177173

**Published:** 2023-05-18

**Authors:** Zhu Suiyi, Wang Jian, Huang Yuhong, Wang Ying, Zhang Yuxin, Qin Jiabao, Liu Jiancong, Yao Jinlu, Ji Meichun

**Affiliations:** ^1^ Colleage of Resource and Environment, Zhongkai University of Agriculture and Engineering, Guangzhou, China; ^2^ Electric Power Research Institute, State Grid Jilin Electric Power Co., Ltd., Changchun, China; ^3^ Guangxi Shenglong Metallurgical Co., Ltd., Fangchenggang, China; ^4^ School of Environment, Tsinghua University, Beijing, China; ^5^ School of Environment, Northeast Normal University, Changchun, China; ^6^ Lversheng (Chongqing) Environmental Technology Co., Ltd., Chongqing, China; ^7^ Taizhou-Shenghe Water Treatment Equipment Manufacturing Co., Ltd., Taizhou, China

**Keywords:** pH adjustment, hydrometallurgy, value-added metals, waste recycling, leachate

## Abstract

pH adjustment was considered a simple step in the hydrometallurgy process, but its complicated operation was ignored in the past. In some industrial applications, the leachate pH was slowly adjusted by a diluted alkaline solution, with the defects of doubling the leachate volume and causing droplet hydrolysis/coagulation. Up to date, promising routes have been developed for rapid pH adjustment, especially in sealed high-temperature/pressure vessels. New routes emerged in some redox/decomposition reactions of nitrate/urea and organics. Such reactions did not start and/or were slow at room temperature but started spontaneously at high temperatures to generate/consume free H^+^. This induced pH adjustment in a rapid and homogeneous way.

## 1 Introduction

Strong-acid extraction is the first step in the hydrometallurgy process to dissolve the valuable metals into leachate from the waste slag/sludge (Yu et al., 2022). After that, the leachate was pretreated by pH adjustment before recycling the valuable metals. It was found that the pH adjustment played a key role in the control of metallic hydrolysis and/or complexation by special reagents, e.g., precipitators (Hoeber and Steinlechner, 2021) and extractants (Flett, 2005). For instance, at pH > 3, the impure Fe can be selectively hydrolyzed and then precipitated as Fe-rich hydroxides, whilst more than 70% Cu is kept in the leachate (Zhu et al., 2020b). The leachate was often adjusted to the optimal pH condition, to vary the functional groups of extracting reagents for selectively complexing valuable metals. It is reported that di-(2-ethylhexyl)phosphoric acid (P204) was an organophosphorus reagent and was used to extract Zn/Ni at pH 2 and 4.5 from leachate (Yuxin et al., 2023), respectively. This indicated the importance of the pH adjustment in the hydrometallurgy process.

**FIGURE 1:**
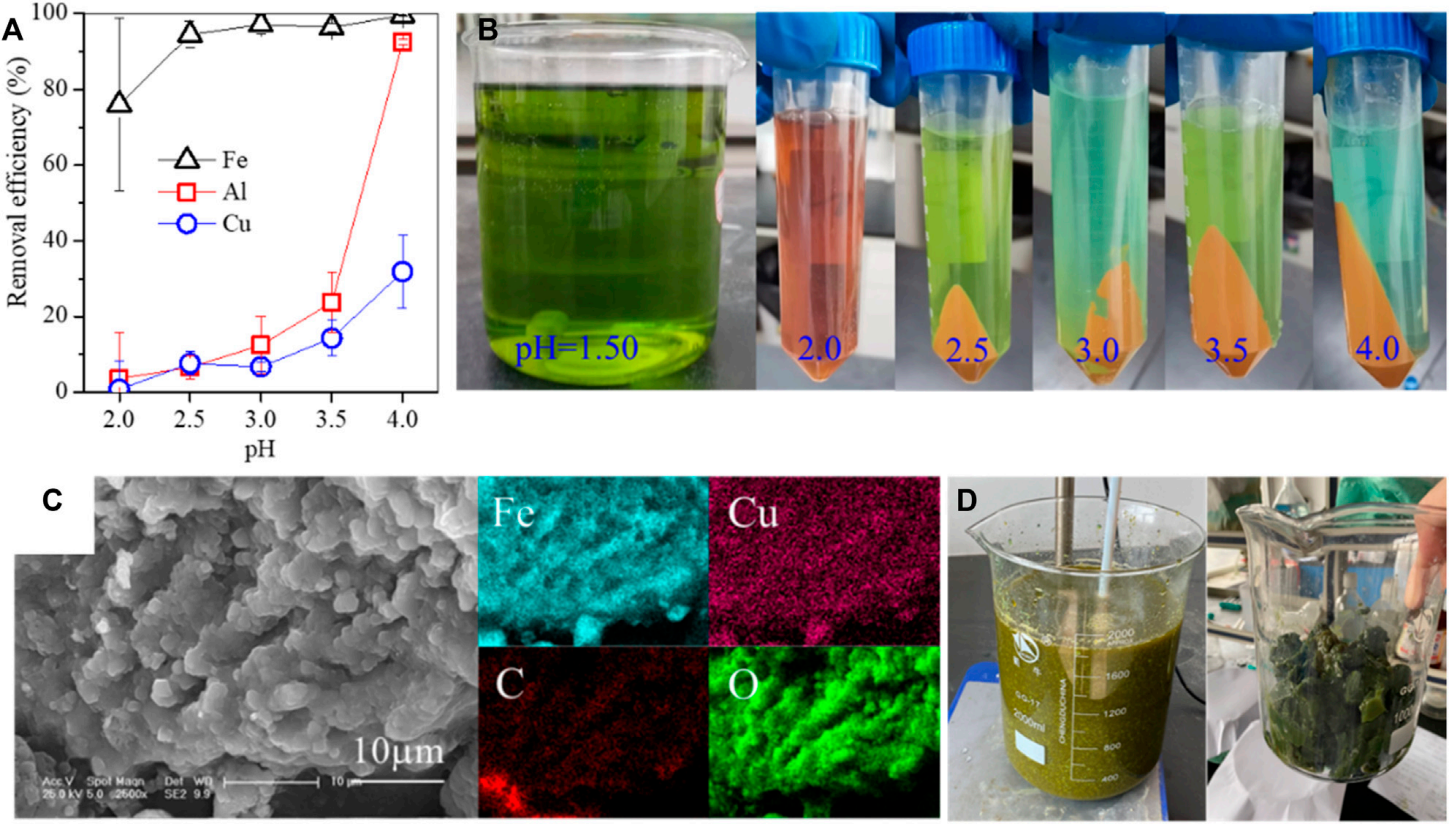
**(A)** Removal efficiencies of Fe/Al/Cu by pH adjustment (the initial Fe/Al/Cu concentrations are 5 g/L, 7.2 g/L, and 6.3 g/L, respectively), **(B)** pictures of Fe/Al precipitation, **(C)** SEM images of Fe-bearing precipitates generated by pH adjustment (Zhu et al., 2020b), and **(D)** pictures of Ni-bearing leachate by pH adjustment.

The pH adjustment is an essential step, but it has three drawbacks in the recycling of valuable metals from leachate. First, the leachate was often adjusted by the diluted alkaline solution, which not only induced extra liquid volume but also decreased the metallic concentration. Accordingly, more waste liquid should be treated after extraction. Second, when the alkaline solution was added to the leachate, the rapid hydrolysis of metallic ions started around the added alkaline to form the metallic hydroxides, resulting in the co-precipitation of metallic ions (Figures 1A, B). This inevitably lowered the recycling efficiency of valuable metals. Third, the alkaline solution was inconveniently added to the sealed high-temperature/pressure vessels. Even though such pH control can be performed at a lab scale via high-speed agitation for a long time, it is not easily replicated in industry.

Apart from the strong-acid leaching, the alkaline leaching was also used for the selective recovery of Zn/Sn from solid waste, especially with the addition of some organics, e.g., sodium dodecyl sulfonate and gelatin. The alkaline leachate was commonly recycled as a metallic sponge via the electrowinning route without the pH adjustment.

Herein, the pH adjustment routes were summarized, especially in the pilot-/industrial-scale hydrometallurgical recycling of valuable metals from acid leachate. The pH adjustment in sealed vessels was introduced, and the related thermochemistry reactions were also analyzed.

## 2 pH adjustment toward acidic conditions

**FIGURE 2:**
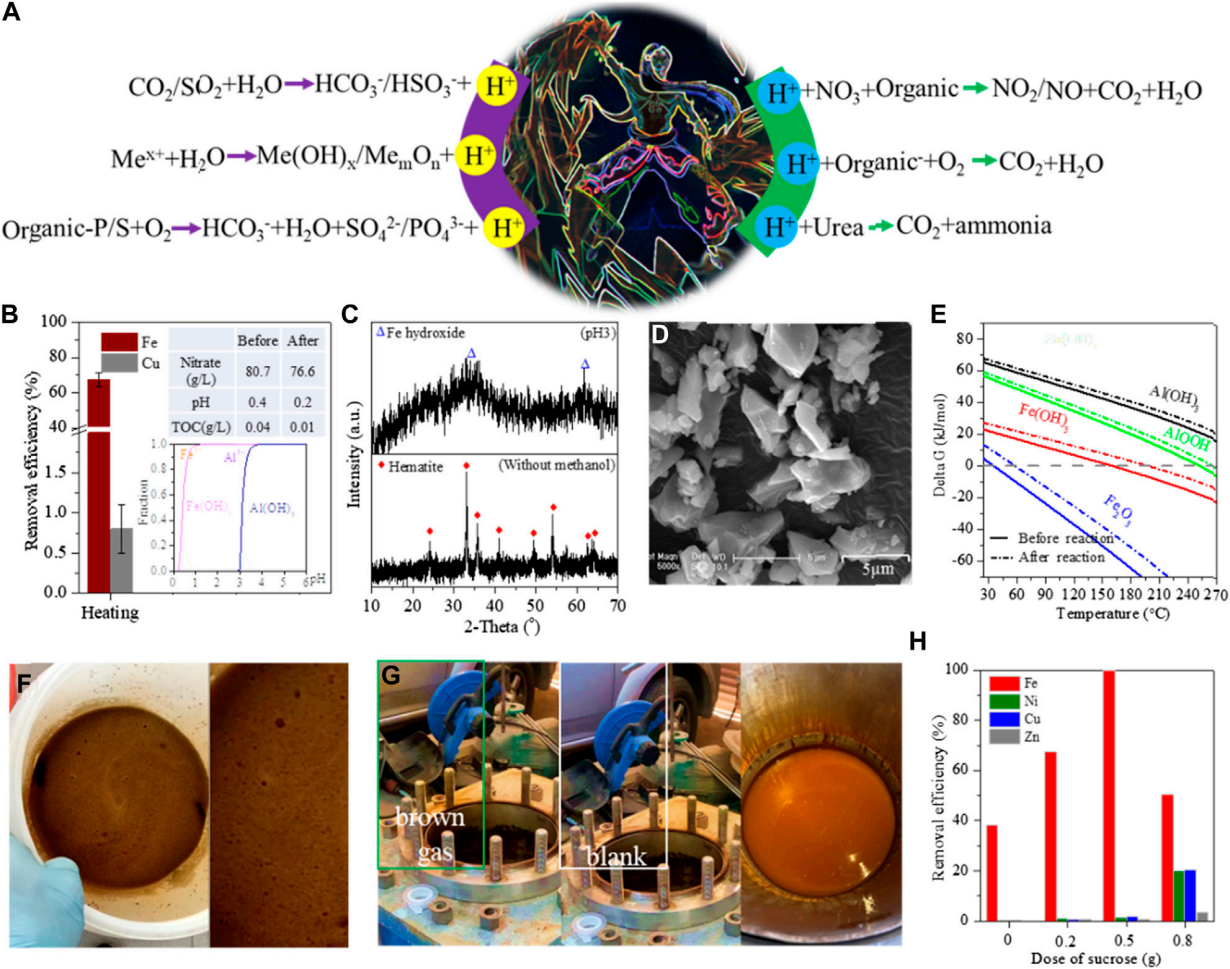
**(A)** Thermochemistry reactions of H^+^ generation and consumption (Xiaobei, 2014), **(B)** removal efficiency of Fe/Cu by hydrothermal treatment (Zhu et al., 2020b), **(C)** XRD patterns of Fe-bearing deposits (Zhu et al., 2020b), **(D)** SEM images of Fe-bearing deposits (Lin et al., 2019), **(E)** fitted plot of Gibbs value versus temperature of Fe/Al hydrolysis (Bian et al., 2022), pictures of **(F)** nitrate reacting with organics and **(G)** emission of NxO in the thermal reaction of nitrate to organics, and **(H)** removal efficiency of Fe/heavy metals after hydrothermal treatment with the addition of sucrose (Yuxin et al., 2023).

Decreasing the leachate pH was easily accomplished by adding strong inorganic and/or organic acids. The added acids were rapidly and uniformly diffused into the leachate after violent agitation. This did not induce hydrolysis and/or co-precipitation of metallic ions. Some reports also showed that the CO_2_/SO_2_ gases were bubbled into neutral or weakly alkaline leachates (Radev et al., 2015) but inappropriately into acidic leachate (Figure 2A). In addition, the pH decrease can be performed in two special ways. First, the hydrolysis of heavy metals and Fe/Al released free H^+^ (Li et al., 2023). This phenomenon was not observed at room temperature but occurred at high temperatures (Figures 2B, C) (Lin et al., 2019; Qu et al., 2021). For example, about 10 g/L Fe was stable in the leachate of Nd-Fe-B waste and removed by 77.6% as hematite (Figure 2D) after heating at 160 C for 10 h, whilst the leachate pH dropped from 0.38 to 0.19 (Lin et al., 2019). Similar hydrolysis of Al at 270°C was also reported to cause a decrease in leachate pH (Figure 2E) (Qu et al., 2021; Li et al., 2023). Second, the organic S and P were oxidized to sulfate and phosphate (Wang et al., 2023), with the generation of free H^+^ into leachate (Figure 2A). This redox reaction was also observed in the wet oxidation of refractory wastewater (Ribeiro et al., 2016).

On an industrial scale, the pH adjustment was also applied in the purification of Zn/Mn from the leachate of zinc blende and/or manganese ore/slag (Yang et al., 2014; Sinha et al., 2022). For instance, the ores were first calcinated to oxidize sulfide and ferrous Fe and then selectively leached by diluted acid and pH adjustment to dissolve Zn/Mn into leachate, alongside few ferrous Fe ions. Conventionally, the obtained leachate was hydrothermally treated at 190°C for 3 h under an oxygen partial pressure of 0.3 MPa to remove 95% Fe with the rest of 2.5 g/L Fe in the treated leachate (Yang et al., 2014). It was noted that the oxidation of ferrous irons commonly consumed an equal amount of free H^+^, but the hydrolysis and crystallization of ferric irons released triple the amount of free H^+^. This steadily dropped the leachate pH and led to the retention of the rest of Fe at a high level.

## 3 How to increase the leachate pH?

Given that the leachate was rich in metallic ions, its pH increase is accompanied by the hydrolysis of metallic ions. Accordingly, polymorphic metallic ions were in the leachate and then easily separated by precipitation (Judge and Azimi, 2020; Zheng et al., 2022), extraction (Yuxin et al., 2023), ion exchange, and so on. As the increase in leachate pH continued, the polymorphic ions were gradually converted to colloids and/or flocs, which then induced co-precipitation of impurities and valuable metals from the leachate.

### 3.1 pH adjustment under atmospheric conditions

As previously described, the leachate pH was adjusted by a diluted alkaline solution to avoid the hydrolysis of metallic ions as insoluble substances (Figures 1A, B). For instance, when the pH was adjusted from 2 to 3, the loss of Cu steadily increased from 0.7% to 6.7%, alongside the removal of 76%–97.1% Fe and 3.6%–12.5% Al. Such an insoluble substance was a polymetallic mixture, in agreement with the loss of valuable metal and the formation of secondary waste (Figure 1C) (Zhu et al., 2020b). In addition, such adjustment was commonly tedious but was also the main step in the hydrometallurgy process. In our pilot-scale experiment, the Ni-rich leachate was adjusted by a diluted sodium hydroxide solution, and then, the aggregated greenish flocs were generated (Figure 1D). Such flocs were suspended in the leachate and cannot be separated by plate-frame pressure filtration. In some industrial processes, the acid leachate was also adjusted by an alkaline solution to maintain a desirable pH range and then extracted by special extracting reagents, e.g., P204 (Yuxin et al., 2023), LIX984, and Cyanex 302 (Soeezi et al., 2020). For instance, the Cu/Ni-rich leachate of the Sarcheshmeh tail was adjusted to pH 2–3 and then extracted by 10% LIX984 to separate Cu, followed by adjusting to pH 4–5 for extracting Ni by 30% Cyanex 302 (Soeezi et al., 2020).

To avoid such drawbacks, some reagents can be considered. For instance, the solid urea was homogeneously dissolved into the leachate at room temperature, whilst leachate pH did not change apparently. After that, the leachate was heated to 80°C and then agitated constantly to decompose urea as ammonia with the capture of free H^+^ (Figure 2A), resulting in an increase in leachate pH (Schaber et al., 2004; Guo et al., 2021). The H^+^ consumption was homogeneous to avoid droplet hydrolysis/flocculation. The urea decomposition was associated with some parameters, e.g., the initial pH value, the reaction temperature, and the urea dosage, so that the corresponding pH could be controlled. Other organics showed a similar decomposition reaction to urea and can also be used in pH adjustment.

Under atmospheric conditions, the redox reaction of nitrite to organics or low-valence metallic ions also consumed the free H^+^ (Figure 2A). Such reactions occurred rapidly, not only to induce the metallic precipitation but also to release yellowish nitrogen dioxide, as shown in the bubbles in Figure 2E. When the nitrite was replaced by oxygen gas, the oxidation of metallic ions also took place at room temperature to consume free H^+^, but that of organics did not occur.

### 3.2 pH adjustment under high-temperature/pressure conditions

Heating the leachate at high temperatures was often accompanied by thermochemistry reactions, including metallic hydrolysis and crystallization, wet oxidation, and pressure dissolution of gases. Especially, the metallic ions were rapidly hydrolyzed at high temperatures and then almost simultaneously converted into highly crystallized minerals. As previously described, the free Fe was hydrothermally hydrolyzed as a hematite block with a drop in leachate pH (Figure 2B) (Lin et al., 2019; Zhu et al., 2020b). Without hydrothermal treatment, the free Fe was stable in the leachate but hydrolyzed as Fe colloid by pH adjustment (Figure 1A), and a portion of Fe colloids was combined as Fe-bearing flocs to cover the free Cu, resulting in the loss of nearly 30% Cu (Zhu et al., 2020b; Jin et al., 2022). In addition, the Fe-bearing flocs showed more coordination sites to adsorb heavy metals compared with hematite (Jianmin, 1994; Qu et al., 2019). At high temperatures, the conversion of Fe colloids to hematite was instantaneous, and thereby, such adsorption drawbacks were effectively repaired. However, with the hydrolysis of free Fe as hematite, free H^+^ was released and accumulated in the leachate, which then reversely inhibited Fe hydrolysis. Other metallic ions showed similar performance to Fe. Therefore, the generated H^+^ should be consumed synchronously to continue the metallic hydrolysis in the high-temperature reaction.

Given that the high temperature was performed in sealed vessels, the following pH adjustment was difficult and can be considered in the following three ways. First, a small number of organics showed a similar structure to urea and decomposed as ammonia to neutralize free H^+^ at a high temperature of >100°C. Second, the redox reaction of nitrite to organics spontaneously occurred at room temperature, but when the nitrite was replaced by nitrate, the corresponding reaction of nitrate to organics was slow at room temperature and started at a temperature of >120°C (Figure 2A) to effectively consume free H^+^. In general, 1 mol of nitrate was reduced by organics with the consumption of 1 mol of free H^+^ (Wieczorek-Ciurowa and Kozak, 1999). Inevitably, yellowish nitrogen dioxide was also generated after opening the vessel (Figure 2G). With the consumption of free H^+^, the metallic hydrolysis continued (Figure 2F). Third, a number of organic acids were used in the leaching of waste slag/sludge and then in the organic anion form. Such organic anions can be removed via wet oxidation with the injection of oxidizing gases, e.g., O_2_ and air, in which the free H^+^ was consumed to steadily increase the leachate pH. When the gas was bubbled into the sealed vessel, the decompression valve was opened to release the generated and unreacted gases so that the leachate and the oxidant were mixed to accelerate the following redox reaction. This operation is similar to the oxygen pressure leaching involved in the selective recycling of valuable metals from ores/minerals (Padilla et al., 2007). It is also found that some impurities of Fe/Al were removed as highly crystallized minerals after hydrothermal treatment, whilst the heavy metals were rarely removed and remained at high concentrations in the leachate (Figures 2B, H).

With such methods, some reagents also entered the leachate and became residues after the reaction. The residues included the generated ammonia, the decomposed organics, and the unreacted nitrate. In our previous research, the rest nitrate was 360 mg/L after reaction with the overdose of methanol (Zhu et al., 2020b) but varied to nearly 30 g/L when the added glucose was inadequate (Bian et al., 2022). It is also found that the rest nitrate and TOC were hundreds of mg/L even at optimal conditions. Such rest impurities should be considered in the following purification of valuable metals from the leachate.

## 4 Conclusion

The pH adjustment was a pretreatment step in the hydrometallurgical recycling of metallic waste. It is tediously operated and also showed defects of doubling leachate volume and inducing hydrolysis/coagulation at lab and industrial scales. At present, the pH adjustment technology focuses on the rapid and uniform diffusion of alkaline species into the leachate, especially in the hydrothermal vessel. Such an adjustment was commonly accompanied by some thermochemistry reactions, including the release of ammonia, the reduction of nitrate, the oxidation of organic anions, and low-valence metallic ions. Such thermochemistry reactions were uncertain, making it difficult to predict the optimal reaction time and the endpoint pH value. However, they made up for the drawbacks of adding an alkaline solution and showed promising application prospects in the separation of targeted metals from the leachate, especially in the sealed high-temperature/pressure system.

## Data Availability

The original contributions presented in the study are included in the article/Supplementary Material; further inquiries can be directed to the corresponding author.
